# Prospective Comparison of [^18^F]FDG and [^18^F]AIF-FAPI-74 PET/CT in the Evaluation of Potentially Resectable Pancreatic Ductal Adenocarcinoma

**DOI:** 10.1007/s11307-024-01950-w

**Published:** 2024-10-04

**Authors:** Won-Gun Yun, Joonhyung Gil, Hongyoon Choi, Youngmin Han, Hye-Sol Jung, Young Jae Cho, Minseok Suh, Wooil Kwon, Yun-Sang Lee, Gi Jeong Cheon, Jin-Young Jang

**Affiliations:** 1 Department of Surgery and Cancer Research Institute, Seoul National University College of Medicine, 101 Daehak-ro, Jongno-gu, Seoul, 03080 Republic of Korea; 2 Department of Nuclear Medicine, Seoul National University College of Medicine, 101 Daehak-ro, Jongno-gu, Seoul, Republic of Korea; 3 Department of Nuclear Medicine, Seoul National University Hospital, 101 Daehak-ro, Jongno- gu, Seoul, Republic of Korea; 4 Institute of Radiation Medicine, Medical Research Center, Seoul National University, Seoul, Republic of Korea; 5 Department of Molecular Medicine and Biopharmaceutical Sciences, Graduate School of Convergence Science and Technology, Seoul National University, Seoul, Republic of Korea; 6 Cancer Research Institute & Institute on Aging, Seoul National University, Seoul, Republic of Korea

**Keywords:** [^18^F]AIF-FAPI-74 PET/CT, [^18^F]FDG PET/CT, Pancreatic ductal adenocarcinoma, Clinical staging, Fibroblast activation protein

## Abstract

**Purpose:**

Accurate clinical staging of potentially resectable pancreatic ductal adenocarcinoma (PDAC) is critical for establishing optimal treatment strategies. While the efficacy of fluorine-18-fluorodeoxyglucose ([^18^F]FDG) positron emission tomography/computed tomography (PET/CT) in clinical staging is unclear, PET/CT detecting fibroblast-activation protein (FAP) expression has recently received considerable attention for detecting various tumors, including PDAC, with high sensitivity. We explored the efficacy of [^18^F]FDG and [^18^F]AIF-FAPI-74 PET/CT in the initial evaluation of potentially resectable PDAC.

**Procedures:**

Between 2021 and 2022, twenty participants with newly diagnosed potentially resectable PDAC were enrolled. After the initial evaluation with pancreatic CT, [^18^F]FDG PET/CT, and [^18^F]AIF-FAPI-74 PET/CT, treatment strategies were determined considering the participant’s general status, clinical staging, and resectability. Pathological information from the surgical specimens was only available in 17 participants who underwent curative-intent surgery. Head-to-head comparisons of quantitative radiotracer uptake and diagnostic performance were performed among imaging modalities.

**Results:**

[^18^F]AIF-FAPI-74 PET/CT showed a significantly higher maximum standardized uptake value than [^18^F]FDG PET/CT did in evaluating primary pancreatic lesions (median [interquartile range]; 12.6 [10.7–13.7] vs. 6.3 [4.8–9.2]; *P* < 0.001). In contrast, [^18^F]AIF-FAPI-74 PET/CT showed a significantly lower mean standardized uptake value than [^18^F]FDG PET/CT did in evaluating background organ (median [interquartile range]) 0.8 [0.7–0.9] vs. 2.6 [2.3–2.7]; *P* < 0.001). In addition, the sensitivity of [^18^F]AIF-FAPI-74 PET/CT in detecting metastatic lymph nodes was higher than that of [^18^F]FDG PET/CT (50.0% vs. 0.0%; *P* = 0.026).

**Conclusion:**

This study demonstrated that [^18^F]AIF-FAPI-74 PET/CT could improve the clinical staging of potentially resectable PDAC.

**Supplementary Information:**

The online version contains supplementary material available at 10.1007/s11307-024-01950-w.

## Introduction

Pancreatic ductal adenocarcinoma (PDAC) is one of the most lethal malignancies with a dismal prognosis, with a 5-year survival rate of approximately 10% [1]. Since surgical resection remains the best chance for long-term survival and cure in patients with PDAC, accurate clinical staging is crucial for improving prognosis [2, 3]. However, because PDAC is frequently accompanied by inflammation and necessitates procedures such as biliary drainage, it is difficult to distinguish the tumorous condition from pancreatitis and estimate the extent of the tumor [4, 5].

Although fluorine-18-fluorodeoxyglucose ([^18^F]FDG) is currently the most frequently used radiotracer for initial tumor staging and recurrence detection in oncological malignancies, the role of [^18^F]FDG positron emission tomography/computed tomography (PET/CT) in PDAC remains unclear. Current recommendations suggest that contrast-enhanced CT or magnetic resonance (MR) should be used as the primary imaging modality prior to treatment in PDAC, with limited recommendations for PET/CT for high-risk patients [6]. Particularly, many studies have revealed the efficacy of [^18^F]FDG PET/CT in identifying extra-pancreatic metastases, yet there is debate over its usefulness in assessing metastatic lymph nodes [7, 8].

Fibroblast-activation protein inhibitors (FAPI) have recently been presented as promising tumor imaging agents that several cancers, including PDAC, can take up [9–13]. Because of the inflammation-induced desmoplastic reaction, PDAC often develops a thick fibrotic stroma with abundant extracellular matrix [14]. The most significant cellular component of the extracellular matrix is cancer-associated fibroblasts (CAF) [15, 16]. As fibroblast-activation protein (FAP) is a pan-marker of CAF, images identifying FAP expression can be considered conceptually appropriate for PDAC. Recently developed FAP-targeting radiotracers showed high sensitivity and specificity for the detection of PDAC lesions in initial studies [17]. However, since studies on the excellence of FAPI PET/CT mostly targeted advanced stages, the efficacy of FAPI PET/CT on early-stage PDAC remains unclear. In addition, [^18^F]AIF-FAPI-74 was used as radiotracers in this study. [^18^F]AIF-FAPI-74 has potential advantages as a tracer, such as a longer half-life and the ability for local supply based on cyclotrons, compared with FAP-targeting tracers with ^68^Ga, which usually require a generator. In contrast to previous studies using ^68^Ga-labeled FAPI, this study focused on potentially resectable PDAC and compared the [^18^F]FDG and [^18^F]AIF-FAPI-74 PET/CT in the same participants. Therefore, we directly compared the diagnostic value and clinical relevance of [^18^F]FDG and [^18^F]AIF-FAPI-74 PET/CT.

## Materials and Methods

### Study Participants

Twenty participants were consecutively recruited for enrollment in this study between October 2021 and September 2022. Written informed consent was obtained from all patients before their inclusion in the study. The eligibility criteria were as follows: (1) age > 19 years, (2) newly diagnosed potentially resectable PDAC by imaging and/or histologic examination, and (3) written informed consent in accordance with institutional and federal regulations. Participants were excluded if they (1) were pregnant, (2) had trouble providing informed consent (3) had serious comorbidities, (4) had undergone previous major abdominal surgery within four weeks of the trial, or (5) were suspected of distant metastasis.

According to the institutional protocol, all included participants underwent pancreatic CT, [^18^F]FDG PET/CT, and blood tests including carbohydrate antigen 19 − 9 (CA 19 − 9, normal range [between 0 and 37 U/mL] and non-secretors [< 2 U/mL on at least 3 examinations]) for initial evaluation; furthermore, [^18^F]AIF-FAPI-74 PET/CT was conducted at the time of diagnosis [18]. After the initial evaluation of PDAC, resectability, staging, and the participant’s overall condition were all carefully considered while choosing the appropriate treatment strategies. Among the 20 participants, 18 (90.0%) underwent surgery and two (10.0%) received chemotherapy. Among the participants who underwent surgery, 1 underwent negative exploration because distant metastasis at the colon mesentery was identified during surgery. Therefore, pathological data from surgical specimens were available for only 17 participants who underwent curative-intent surgery (Fig. S1).

### Radiosynthesis of [18F]AlF-FAPI-74

Radio thin layer chromatography instant thin layer chromatography-silica gel plates were obtained from the Pall Company (New York, USA). A Bioscan AR-2000 thin layer chromatography Imaging Scanner (Washington, DC, USA) was used to perform the Radio thin layer chromatography scan. A Gilson High Pressure Liquid Chromatography system (Middleton, USA) was used for purification or the purity check of [^18^F]AlF-FAPI-74. C18 and QMA Sep-Pak cartridges were obtained from Waters (Milford, USA), and [^18^F]Fluoride was produced by the ^18^O(p, n)^18^F reaction on ^18^O-enriched (97%) water using a 16.5 MeV proton beam generated by a GE PETtrace™ 800 cyclotron (Chicago, USA). The precursor of [^18^F]AlF-FAPI-74 was supplied by SOFIE Biosciences (Dulles, USA).

[^18^F]AlF-FAPI-74 was prepared from its precursor (S)-(4-carboxymethyl-7-{2-[4-(3-{4-[2-(2-cyano-pyrrolidin-1-yl)-2-oxo-ethylcarbamoyl]-quinolin-6-yloxy]-propyl}-piperazin-1-yl)-acetic acid based on the previously reported method with the slight modification [19]. Briefly, an aqueous solution of [^18^F]fluoride in ^18^O-enriched water was captured on QMA light Sep-Pak cartridge, and [^18^F]fluoride on the cartridge was eluted with 0.9% sodium chloride solution (0.6 mL) and methyl cyanide (0.9 mL) and reacted with aluminum chloride (0.8 µg, 6 nmol in pH 3.9, 0.5 M acetate buffer (10 µL) for 5 min at 50 °C. FAPI-74 (74 µg, 0.1 µmol) in water (74 µL) was added to the reaction mixture and reacted for 10 min at 100 °C. The reaction mixture was purified with C18 Sep-Pak cartridges to give [^18^F]AlF-FAPI-74 (radiochemical purity: 99.5%; radiochemical yield: 25.3 ± 6.3%; molar activity: 110 ± 34.4 GBq/µmol).

### [18F]FDG and [18F]AIF-FAPI-74 PET/CT Protocol

Prior to [^18^F]FDG PET/CT imaging, the participants fasted for at least 8 h and their blood glucose levels were measured. Participants received 5.18 MBq/kg [^18^F]FDG when their blood glucose level was less than 200 mg/dL. After voiding, a PET/CT scan was performed 60 min after [^18^F]FDG injection. For [^18^F]AIF-FAPI-74 PET/CT, participants received 185 MBq of [^18^F]AIF-FAPI-74 PET/CT scan were performed 120 min after radiotracer injection.

We used 3 dedicated PET/CT scanners (Biograph TruePoint 40, Biograph mCT 40, and Biograph mCT 64; Siemens Healthineers, Erlangen, Germany). CT scan was performed from the cranial base to the proximal thigh without contrast enhancement. The following parameters were used for the scan: 120 kVp; matrix size, 512 × 512; slice thickness, 5 mm (TruePoint 40) or 3 mm (mCT 40, mCT 60). Subsequently, PET emission scans were performed at 7 to 9 bed positions (2 min/bed, TruePoint; 1 min/bed, mCT). Attenuation-corrected PET images were reconstructed using the following parameters: TruePoint, iterations, 2; subsets, 21; 3-mm full-width half-maximum Gaussian filter; matrix, 168 × 168; mCT, iterations, 2; subsets, 21; 5-mm full-width half-maximum Gaussian filter; and matrix, 200 × 200.

### Image Interpretation

PET/CT images were analyzed using an image analysis software (Syngo.via VB20, Siemens Healthineers). Semi-quantitative and qualitative methods were used to analyze the images. The semi-quantitative analysis included measurement of PET parameters such as maximum and mean standardized uptake values (SUV_max_, SUV_mean_) and total lesion activity (TLA; total lesion glycolysis in [^18^F]FDG PET/CT; total lesion FAP expression in [^18^F]AIF-FAPI-74 PET/CT). The SUV threshold with 40% of SUV_max_ was used to measure volumetric parameters. To measure the background activity, the volume of interest was drawn on the right posterior side of the liver with a 3 cm diameter sphere. In qualitative analysis, lymph nodes with higher activity than that of the regional background were regarded as positive. Three nuclear medicine physicians (two senior resident physicians, and one experienced board-certified physician) evaluated the lymph node positivity on [^18^F]FDG and [^18^F]AIF-FAPI-74 PET/CT and obtained consensus data. On pancreatic CT, lymph nodes ≥ 10 mm were defined as pathological lymph nodes.

### Statistical Analysis

All statistical analyses were performed using the R software, version 4.2.2 (R Foundation for Statistical Computing). Categorical variables are expressed as numbers with percentages, and continuous variables are expressed as medians with interquartile ranges (IQR). Differences in the SUV between [^18^F]FDG and [^18^F]F-FAPI-74 PET/CT were evaluated using the Wilcoxon signed-rank test (skewed variables). The sensitivity, specificity, accuracy, positive predictive value, and negative predictive value of [^18^F]FDG and [^18^F]F-FAPI-74 PET/CT were calculated by comparing the predictive nodal status with the postoperative histology in participants who underwent curative-intent surgery. The sensitivities and specificities of the imaging modalities were compared using McNemar’s test. Pearson’s correlation was used to determine the association between CA 19 − 9 and TLA, and Fisher’s Z-transformation was performed to compare the correlation coefficients. All P-values were two-sided, and *P* < 0.05 was considered statistically significant.

## Results

###  Participant Characteristics

The baseline characteristics of the participants are summarized in Table 1. The median (IQR) age was 69.0 (66.0–77.3) years old. There were eight men (40.0%) and 12 women (60.0%). Of these participants, four (20.0%) were identified as CA 19 − 9 non-secretors, and 13 (65.0%) had elevated CA 19 − 9 levels. Among participants who underwent curative-intent surgery, 10 (58.8%) had lymph node metastases, and 14 (82.4%) were diagnosed at a relatively early stage (pathological stage I or II) based on the American Joint Committee on Cancer 8th edition.

**Table 1:** Baseline characteristics of study participants

Variables	Value
Number of participants	20
Age, median, interquartile range, y (*N* = 20)	69.0 (66.0–77.3)
Sex (*N* = 20)	
Male	8 (40.0%)
Female	12 (60.0%)
ASA classification (*N* = 20)	
1 / 2	19 (95.0%)
3 / 4	1 (5.0%)
Location (*N* = 20)	
Head	6 (30.0%)
Body/Tail	14 (70.0%)
CA 19 − 9, U/mL (*N* = 20)	
Normal	3 (15.0%)
Elevated	13 (65.0%)
Non-secretors	4 (20.0%)
Treatment strategy (*N* = 20)	
Chemotherapy	2 (10.0%)
Surgery	18 (90.0%)
Surgery (*N* = 18)^a^	
PD/PPPD	5 (27.8%)
DP	12 (66.7%)
Negative exploration	1 (5.5%)
Pathological T stage (*N* = 17)^b^	
T1	3 (17.6%)
T2	12 (70.6%)
T3	2 (11.8%)
T4	0 (0.0%)
Pathological N stage (*N* = 17)^b^	
N0	7 (41.2%)
N+	10 (58.8%)
Pathological AJCC stage (*N* = 17)^b^	
1	5 (29.4%)
2	9 (53.0%)
3	3 (17.6%)
4	0 (0.0%)

^a^Participants who underwent surgery^b^Participants who underwent curative-intent surgery*ASA* American Society of Anesthesiologists, *CA 19 − 9* carbohydrate antigen 19 − 9, *PD* pancreaticoduodenectomy, *PPPD* pylorus-preserving pancreaticoduodenectomy, *DP* distal pancreatectomy

### Evaluation of Primary Pancreatic Lesions

**Fig. 1:**
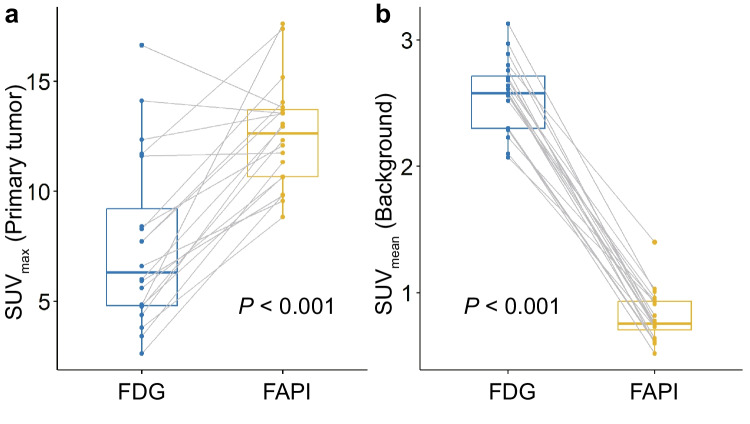
Differences of radiotracer uptake of (**a**) primary pancreatic lesions and (**b**) background organs between [^18^F]FDG and [^18^F]AIF-FAPI-74 PET/CT

The results showed that both [^18^F]FDG and [^18^F]AIF-FAPI-74 PET/CT could detect all 20 primary pancreatic lesions with a positive detection rate of 100%. We further compared the SUV_max_ of the primary pancreatic lesions and the SUV_mean_ of the background between the [^18^F]FDG and [^18^F]AIF-FAPI-74 PET/CT images. In primary pancreatic lesions, [^18^F]AIF-FAPI-74 PET/CT showed higher median [IQR] SUV_max_ than did [^18^F]FDG PET/CT (12.6 [10.7–13.7] vs. 6.3 [4.8–9.2], *P* < 0.001, Fig. 1a). In the background organ, as opposed to primary pancreatic lesions, [^18^F]AIF-FAPI-74 PET/CT showed a lower median [IQR] SUV_mean_ than [^18^F]FDG PET/CT (0.8 [0.7–0.9] vs. 2.6 [2.3–2.7], *P* < 0.001, Fig. 1b).

**Fig. 2:**
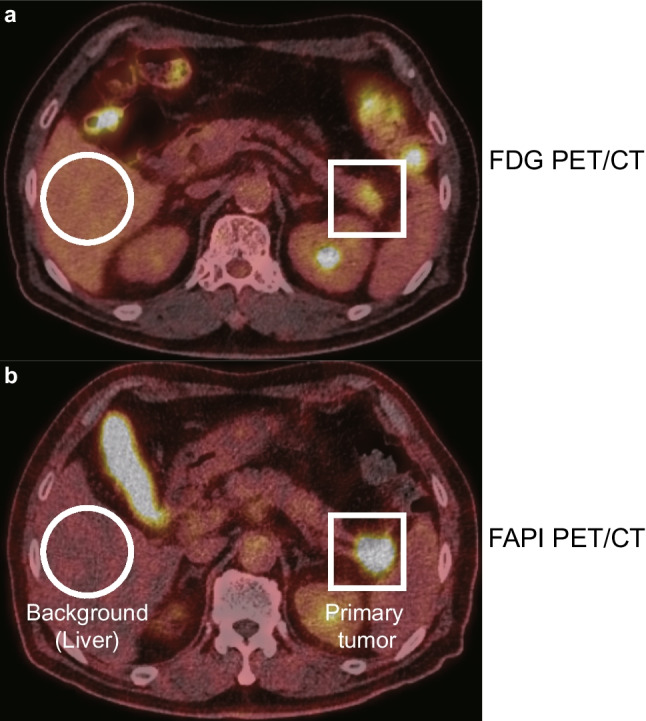
(**a**) [^18^F]FDG and (**b**) [^18^F]AIF-FAPI-74 PET/CT images of a 72-year-old man with newly diagnosed pancreatic tail cancer for tumor staging

An example of measuring uptake in primary pancreatic lesions and the background are presented in Fig. 2. The primary pancreatic lesions were delineated more clearly, and the radiotracer uptake in background organs, such as the liver, was lower on [^18^F]AIF-FAPI-74 PET/CT than on [^18^F]FDG PET/CT. Although there was no difference in the detection rates of primary pancreatic lesions between [^18^F]FDG and [^18^F]AIF-FAPI-74 PET/CT, a distinct difference in radiotracer uptake implies that [^18^F]AIF-FAPI-74 PET/CT is more specific than [^18^F]FDG PET/CT.

### Diagnostic Performance of [18F]FDG and [18F]AIF-FAPI-74 PET/CT in Nodal Metastasis

Among the 17 participants who underwent curative-intent surgery, 10 had lymph node metastases in the final pathology report. Table 2 shows the performance of the imaging modalities in detecting metastatic lymph nodes. [^18^F]AIF-FAPI-74 PET/CT showed significantly higher sensitivity than did [^18^F]FDG PET/CT (50.0% [5/10] vs. 0.0% [0/10]; *P* = 0.026). [^18^F]AIF-FAPI-74 PET/CT showed higher sensitivity than did pancreatic CT, which is the standard pretreatment imaging, but this did not reach statistical significance (50.0% [5/10] vs. 30.0% [3/10]; *P* = 0.655). The specificity was 100.0% (0/7) for [^18^F]FDG and [^18^F]AIF-FAPI-74 PET/CT and 85.7% (6/7) for pancreatic CT. Additionally, [^18^F]AIF-FAPI-74 PET/CT showed the highest accuracy, positive predictive value, and negative predictive value for detecting metastatic lymph nodes compared to [^18^F]FDG PET/CT or pancreatic CT.

**Table 2:** Comparison of performance in predicting nodal status among imaging modalities

	Pancreatic CT		[^18^F]FDG PET/CT		[^18^F]AIF-FAPI-74 PET/CT	
	cN+	cN0	cN+	cN0	cN+	cN0
**pN+**	3	7	0	10	5	5
**pN0**	1	6	0	7	0	7
Sensitivity	30.0%		0.0%		50.0%	
Specificity	85.7%		100.0%		100.0%	
Accuracy	52.9%		41.2%		70.6%	
PPV	75.0%		Not applicable		100.0%	
NPV	46.2%		41.2%		58.3%	
*P*-value	0.046^a^			0.026^b^		
	0.655^c^					

^a^Comparison between pancreatic CT and [^18^F]FDG PET/CT^b^Comparison between [^18^F]FDG PET/CT and [^18^F]AIF-FAPI-74 PET/CT^c^Comparison between pancreatic CT and [^18^F]AIF-FAPI-74 PET/CT*PPV* positive predictive value, *NPV* negative predictive value

**Fig. 3:**
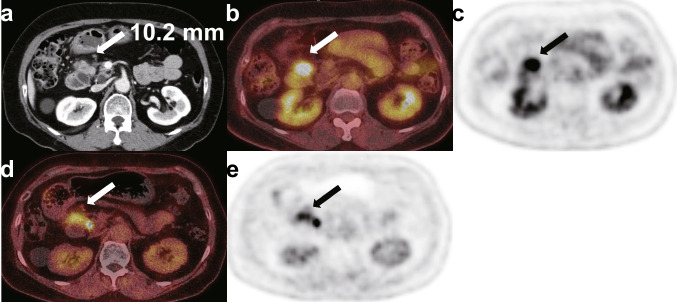
Preoperative images of a 66-year-old woman with newly diagnosed pancreatic head cancer for tumor staging. A pathological lymph node at station 17 larger than 10 mm on (white arrow in **a**) pancreatic CT did not show distinguishable radiotracer uptake in (white arrow in **b**, black arrow in **c**) [^18^F]FDG PET/CT but showed distinguishable radiotracer uptake in (white arrow in **d**, black arrow in **e**) [^18^F]AIF-FAPI-74 PET/CT. The SUV intensity-scale of the PET/CT images was set from 0 to 5

An example case of this is shown in Fig. 3. Pancreatic CT, [^18^F]FDG, and [^18^F]AIF-FAPI-74 PET/CT images show a lymph node at station 17 in a 66-year-old woman with newly diagnosed pancreatic head cancer. A 10.2 mm sized pathological lymph node exhibiting [^18^F]AlF-FAPI-74 uptake was detected on preoperative imaging, which was subsequently confirmed to be a metastasis after postoperative evaluation. However, this lymph node did not show distinct uptake from the surrounding area on [^18^F]FDG PET/CT.

**Fig. 4:**
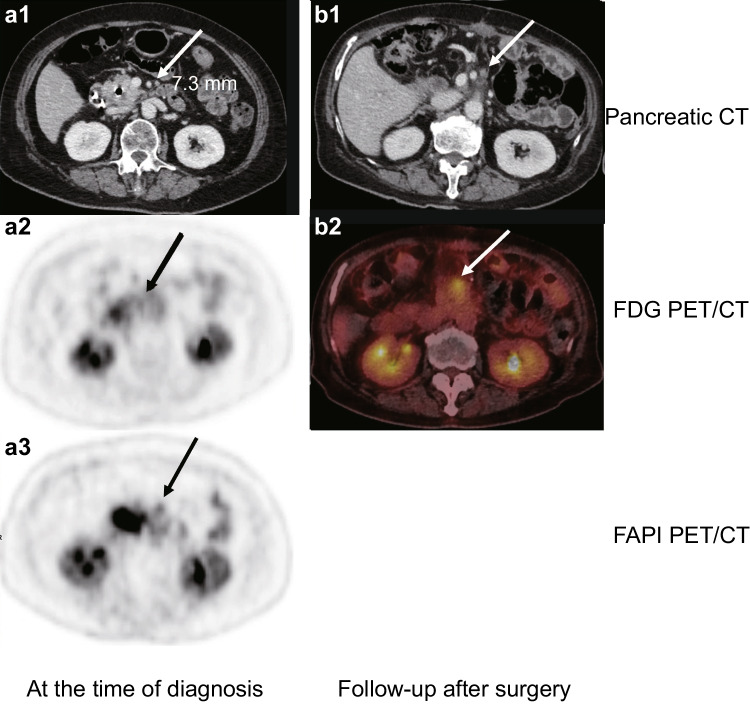
Preoperative and follow-up images of a 72-year-old woman with newly diagnosed pancreatic head cancer. A 7.3 mm-sized lymph node at station 14 on (white arrow in **a1**) preoperative CT did not show distinguishable radiotracer uptake in (black arrow in **a2**) [^18^F]FDG PET/CT but showed distinguishable radiotracer uptake in (black arrow in **a3**) [^18^F]AIF-FAPI-74 PET/CT. After surgery, remaining lymph node at station 14 was observed in (white arrow in **b1**) postoperative 3 months CT, and local recurrence around superior mesenteric artery was observed in (white arrow in **b2**) postoperative 10 months [^18^F]FDG PET/CT

Another example case is shown in Fig. 4. Pancreatic CT, [^18^F]FDG, and [^18^F]AIF-FAPI-74 PET/CT images revealed a lymph node at station 14 near the superior mesenteric artery in a 72-year-old woman with a newly diagnosed pancreatic head cancer. A 7.3 mm-sized indeterminate lymph node on preoperative pancreatic CT did not show distinct radiotracer uptake from the surrounding areas on [^18^F]FDG PET/CT; however, there was distinguishable uptake on [^18^F]AIF-FAPI-74 PET/CT. At the time of diagnosis, the lymph node at station 14 was not identified and it was not generally included in the extent of standard lymph node dissection during pancreaticoduodenectomy; therefore, the lymph node was observed in postoperative 3 months follow-up pancreatic CT. Local recurrence around the superior mesenteric artery eventually occurred 10 months after surgery following adjuvant chemotherapy, as the size progressively increased and metabolic activity was observed.

### Correlation between CA 19 − 9 and Parameters of [18F]FDG and [18F]AIF-FAPI-74 PET/CT

**Fig. 5:**
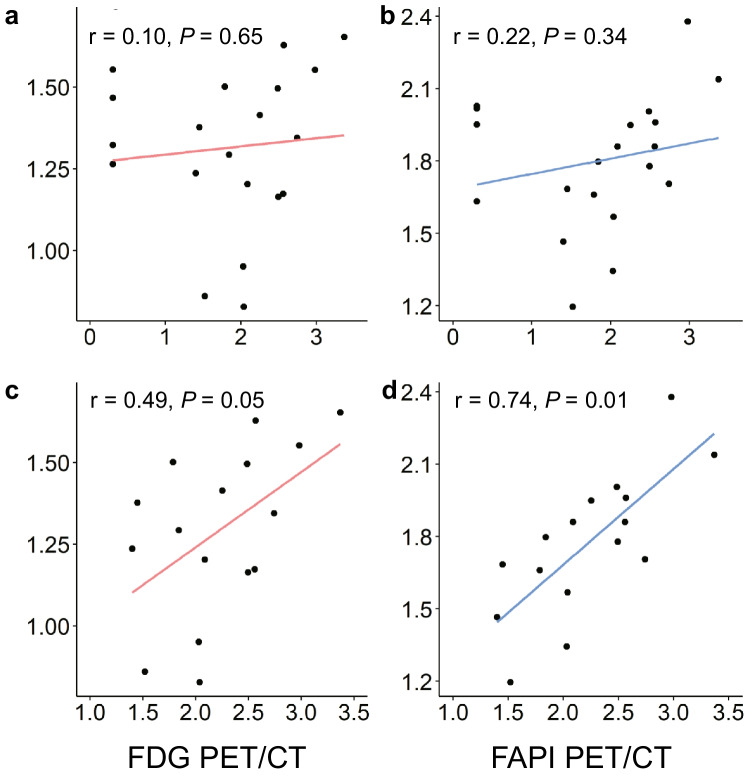
Correlation between log transformed CA 19 − 9 (X-axis) and log transformed total lesion activity (Y-axis) of (**a**) [^18^F]FDG and (**b**) [^18^F]AIF-FAPI-74 PET/CT in total 20 participants. Correlation between log transformed CA 19 − 9 (X-axis) and log transformed total lesion activity (Y-axis) of (**c**) [^18^F]FDG and (**d**) [^18^F]AIF-FAPI-74 PET/CT in 16 participants after excluding CA 19 − 9 non-secretors

To evaluate the clinical relevance of the PET/CT parameters, the correlation between CA 19 − 9, which is a representative tumor marker in PDAC, and the TLA of primary pancreatic lesions, a representative indicator of tumor burden, was evaluated. In 20 participants including four CA 19 − 9 non-secretors, a correlation between CA 19 − 9 and TLA was not observed in either [^18^F]FDG or [^18^F]AIF-FAPI-74 PET/CT images (r_1_all_ = 0.10, *P* = 0.65 for [^18^F]FDG PET/CT and r_2_all_ = 0.22, *P* = 0.34 for [^18^F]AlF-FAPI-74 PET/CT, Fig. 5a–b). However, when four CA 19 − 9 non-secretors were excluded from the analysis, CA 19 − 9 was significantly correlated with TLA in both [^18^F]FDG (r_1_secretors_ = 0.49, *P* = 0.05) and [^18^F]AlF-FAPI-74 PET/CT images (r_2_secretors_ = 0.74, *P* = 0.01; Fig. 5c–d). The correlation coefficient of TLA measured on [^18^F]AlF-FAPI-74 PET/CT was higher than that measured on [^18^F]FDG, although the statistical comparison of the correlation coefficients was not significant (r_1_secretors_ = 0.49 vs. r_2_secretors_ = 0.74, Z = -1.06, *P* = 0.29).

## Discussion

In PDAC, [^18^F]FDG PET/CT is recommended for high-risk patients as an adjunct to pancreatic CT [6]. FAPI, an imaging agent for the visualization of the tumor stroma, represents a promising alternative to FDG [9]. This study showed that PDAC is a highly FAPI-avid tumor, and [^18^F]AIF-FAPI-74 PET/CT showed some superiority to [^18^F]FDG PET/CT in the detection of primary pancreatic cancer and metastatic lymph nodes. In addition, we found that the TLA of [^18^F]AIF-FAPI-74 PET/CT had a stronger correlation with CA 19 − 9, compared to that of [^18^F]FDG PET/CT. Therefore, PET/CT imaging with [^18^F]AIF-FAPI-74 may be advantageous for the clinical management of patients with potentially resectable PDAC.

Because surgical resection remains the only treatment with curative potential for PDAC, optimizing the extent of surgical resection is very important. The extent of pancreatectomy can be easily determined. However, the extent of lymph node dissection has occasionally been debated. Although some randomized controlled trials reported that extended lymph node dissection does not improve oncologic outcomes compared with standard lymph node dissection in performing pancreaticoduodenectomy, pathologic examination should be performed when pathological lymph nodes around the superior mesenteric artery, celiac axis, and aorta are suspected in preoperative evaluation [20–22]. Particularly, it is known that adding [^18^F]FDG PET/CT to pancreatic CT can improve the sensitivity of detecting distant metastatic lesions, but neither [^18^F]FDG PET/CT nor pancreatic CT showed good performance at detecting metastatic lymph nodes [8]. This study showed that [^18^F]AIF-FAPI-74 PET/CT had superior efficacy to [^18^F]FDG PET/CT or pancreatic CT in the detection of metastatic lymph nodes. In particular, the examples in Figs. 3 and 4 illustrate the good diagnostic accuracy of [^18^F]AIF-FAPI-74 PET/CT for metastatic lymph nodes, regardless of whether the lymph nodes were larger or smaller than 10 mm. Small lymph nodes may be underestimated on PET scans due to the partial volume effect, but the higher avidity of [^18^F]AIF-FAPI-74 compared to [^18^F]FDG allows for the evaluation of small lymph nodes with PET scans. Additionally, Zhang et al. (2022) reported that [^68^Ga]Ga-DOTA-FAPI-04 PET/MR might be better than [^18^F]FDG PET/CT in the detection of suspicious lymph node metastasis in PDAC [23]. However, this paper was a comparison between PET/MR and PET/CT, and most of the participants were in advanced stages (stage III or IV). Our study has strengths in that it is a comparison between PET/CT scans, and most of the participants (17/20, 85.0%) were operable.

Numerous research on the clinical relevance of [^18^F]FDG PET/CT in addition to diagnostic efficacy have been undertaken over the past few decades. Moon et al. (2022) and Lee et al. (2014) reported that SUV_max_ and TLA on preoperative [^18^F]FDG PET/CT showed good performance on survival prediction in surgically treated PDAC [24, 25]. Lee et al. (2021) also reported that a reduction in radiotracer uptake of [^18^F]FDG PET/CT after neoadjuvant chemotherapy was associated with improved survival outcomes in PDAC [26]. This study found that FAP expression is more PDAC-specific than glucose metabolism by comparing the tumor-to-background ratio in [^18^F]FDG and [^18^F]AIF-FAPI-74 PET/CT. Kawase et al. (2015) reported that moderate or strong stromal FAP staining intensity in immunohistochemistry was associated with poor prognosis [27]. FAP is mainly expressed in CAFs, and especially FAP-expressing CAFs are known to secrete CXCL12 to provide an immunosuppressive milieu [15]. These findings raise the possibility of using FAP expression as an indicator for predicting the effectiveness of chemotherapy or immunotherapy rather than just a prognostic factor. Currently, CA 19 − 9 is used as the most representative tumor marker in PDAC; however, approximately 5–10% of individuals are still Lewis antigen-negative, with the scarce secretion of CA 19 − 9. The parameters provided by [^18^F]AIF-FAPI-74 PET/CT can be PDAC-specific markers that can replace CA 19 − 9, especially in CA 19 − 9 non-secretors. However, this possibility needs to be verified in a large-scale prospective study.

There is no internationally agreed-upon protocol for FAPI PET/CT. Therefore, the radiotracer and time taken to acquire images after injecting the radiotracer vary among medical centers. ^68^Ga-labeled FAPI compounds have been widely used in other studies; however, this study used ^18^F-labeled radiotracers. The advantage of using ^18^F-labeled compounds is the availability of commercial supplements without needing a generator, unlike ^68^Ga-labeled compounds which require a generator and have a shorter half-life of 68 minutes. The half-life of ^18^F-labeled compounds is 110 min, which makes it possible to acquire delayed PET/CT images and provides additional clinical options [19]. Indeed, although only 120-minute [^18^F]AIF-FAPI-74 PET/CT images were used in this study, we also obtained 60-minute images. The 60-minute [^18^F]AIF-FAPI-74 PET/CT images also showed better diagnostic performance than did [^18^F]FDG PET/CT, but the 120-minute images were better. For primary pancreatic lesions, 120-minute [^18^F]AIF-FAPI-74 PET/CT (16.1 [13.1–18.2]) showed significantly higher median [IQR] tumor-to-background ratio than 60-minute [^18^F]AIF-FAPI-74 PET/CT (13.8 [12.1–15.3]; *P* < 0.001, Fig. S2). Pang et al. (2021) also suggested that delayed FAPI PET/CT images were more suitable for distinguishing inflammation from tumorous conditions in PDAC [28]. However, we found that both 60 and 120-minute [^18^F]AIF-FAPI-74 PET/CT showed physiologic or non-malignant radiotracer accumulation in the biliary tract. Of the 20 participants, 19 (95.0%) showed radiotracer accumulation in the biliary tract. This could be a pitfall in interpreting the [^18^F]AIF-FAPI-74 PET/CT images of biliary tract cancers. Therefore, further research is needed to establish an optimal FAPI PET/CT imaging protocol for carcinoma type.

Our study has a few limitations. First, our study sample included a relatively small number of participants; further studies are warranted to confirm these findings. Second, although there was a considerable correlation between CA 19 − 9 and TLA on [^18^F]AIF-FAPI-74 PET/CT, the survival outcome according to TLA could not be assessed because of the insufficient follow-up period. Therefore, further studies should be based on larger sample sizes and longer follow-up periods.

## Conclusions

Our results showed that [^18^F]AIF-FAPI-74 PET/CT was superior to [^18^F]FDG PET/CT in detecting primary tumors and metastatic lymph nodes in potentially resectable PDAC. Additionally, the TLA from [^18^F]AIF-FAPI-74 PET/CT correlated well with CA 19 − 9. These findings suggest that [^18^F]AIF-FAPI-74 PET/CT is a potential alternative to [^18^F]FDG PET/CT for initial evaluation of potentially resectable PDAC.

## Supplementary Information

Below is the link to the electronic supplementary material.

ESM1 (MOESM1 1.21 MB)

## Data Availability

The data that support the findings of this study are available on request from the corresponding author.

## References

[1] Siegel RL, Miller KD, Fuchs HE, Jemal A (2022) Cancer statistics, 2022. CA Cancer J Clin 72:7–33 35020204 10.3322/caac.21708

[2] Kang MJ, Yun EH, Jung KW, Park SJ (2022) Incidence, mortality and survival of gallbladder, extrahepatic bile duct, and pancreatic cancer using Korea central cancer registry database: 1999–2019. Ann Hepatobiliary Pancreat Surg 26:220–228 35909086 10.14701/ahbps.22-041 PMC9428428

[3] Park W, Chawla A, O’Reilly EM (2021) Pancreatic Cancer: a review. JAMA 326:851–862 34547082 10.1001/jama.2021.13027 PMC9363152

[4] Wolske KM, Ponnatapura J, Kolokythas O, Burke LMB, Tappouni R, Lalwani N (2019) Chronic pancreatitis or pancreatic tumor? A problem-solving Approach. Radiographics 39:1965–1982 31584860 10.1148/rg.2019190011

[5] Schima W, Bohm G, Rosch CS, Klaus A, Fugger R, Kopf H (2020) Mass-forming pancreatitis versus pancreatic ductal adenocarcinoma: CT and MR imaging for differentiation. Cancer Imaging 20:52 32703312 10.1186/s40644-020-00324-z PMC7376657

[6] Tempero MA, Malafa MP, Al-Hawary M et al (2021) Pancreatic adenocarcinoma, Version 2.2021, NCCN Clinical Practice guidelines in Oncology. J Natl Compr Canc Netw 19:439–457 33845462 10.6004/jnccn.2021.0017

[7] Farma JM, Santillan AA, Melis M et al (2008) PET/CT fusion scan enhances CT staging in patients with pancreatic neoplasms. Ann Surg Oncol 15:2465–2471 18551347 10.1245/s10434-008-9992-0

[8] Wang Z, Chen JQ, Liu JL, Qin XG, Huang Y (2013) FDG-PET in diagnosis, staging and prognosis of pancreatic carcinoma: a meta-analysis. World J Gastroenterol 19:4808–4817 23922481 10.3748/wjg.v19.i29.4808 PMC3732856

[9] Loktev A, Lindner T, Mier W et al (2018) A tumor-imaging Method Targeting Cancer-Associated fibroblasts. J Nucl Med 59:1423–1429 29626120 10.2967/jnumed.118.210435 PMC6126438

[10] Giesel FL, Kratochwil C, Lindner T et al (2019) (68)Ga-FAPI PET/CT: Biodistribution and preliminary Dosimetry Estimate of 2 DOTA-Containing FAP-Targeting agents in patients with various cancers. J Nucl Med 60:386–392 30072500 10.2967/jnumed.118.215913 PMC6424229

[11] Kratochwil C, Flechsig P, Lindner T et al (2019) (68)Ga-FAPI PET/CT: Tracer Uptake in 28 different kinds of Cancer. J Nucl Med 60:801–805 30954939 10.2967/jnumed.119.227967 PMC6581228

[12] Ding J, Qiu J, Hao Z et al (2023) Prognostic value of preoperative [(68) Ga]Ga-FAPI-04 PET/CT in patients with resectable pancreatic ductal adenocarcinoma in correlation with immunohistological characteristics. Eur J Nucl Med Mol Imaging 10.1007/s00259-022-06100-4 36695823

[13] Lang M, Spektor AM, Hielscher T et al (2023) Static and dynamic (68)Ga-FAPI PET/CT for the detection of Malignant Transformation of Intraductal Papillary Mucinous Neoplasia of the pancreas. J Nucl Med 64:244–251 35906094 10.2967/jnumed.122.264361 PMC9902850

[14] Pandol S, Edderkaoui M, Gukovsky I, Lugea A, Gukovskaya A (2009) Desmoplasia of pancreatic ductal adenocarcinoma. Clin Gastroenterol Hepatol 7:S44–47 19896098 10.1016/j.cgh.2009.07.039 PMC4573641

[15] Liu T, Han C, Wang S et al (2019) Cancer-associated fibroblasts: an emerging target of anti-cancer immunotherapy. J Hematol Oncol 12:86 31462327 10.1186/s13045-019-0770-1 PMC6714445

[16] Zhang T, Ren Y, Yang P, Wang J, Zhou H (2022) Cancer-associated fibroblasts in pancreatic ductal adenocarcinoma. Cell Death Dis 13:897 36284087 10.1038/s41419-022-05351-1 PMC9596464

[17] Rohrich M, Naumann P, Giesel FL et al (2021) Impact of (68)Ga-FAPI PET/CT imaging on the Therapeutic Management of primary and recurrent pancreatic ductal adenocarcinomas. J Nucl Med 62:779–786 33097632 10.2967/jnumed.120.253062 PMC8729866

[18] Parra-Robert M, Santos VM, Canis SM, Pla XF, Fradera JMA, Porto RM (2018) Relationship between CA 19.9 and the Lewis phenotype: options to improve diagnostic efficiency. Anticancer Res 38:5883–5888 30275214 10.21873/anticanres.12931

[19] Giesel FL, Adeberg S, Syed M et al (2021) FAPI-74 PET/CT using either (18)F-AlF or Cold-Kit (68)Ga labeling: Biodistribution, Radiation Dosimetry, and Tumor Delineation in Lung Cancer patients. J Nucl Med 62:201–207 32591493 10.2967/jnumed.120.245084 PMC8679591

[20] Jang JY, Kang MJ, Heo JS et al (2014) A prospective randomized controlled study comparing outcomes of standard resection and extended resection, including dissection of the nerve plexus and various lymph nodes, in patients with pancreatic head cancer. Ann Surg 259:656–664 24368638 10.1097/SLA.0000000000000384

[21] Jang JY, Kang JS, Han Y et al (2017) Long-term outcomes and recurrence patterns of standard versus extended pancreatectomy for pancreatic head cancer: a multicenter prospective randomized controlled study. J Hepatobiliary Pancreat Sci 24:426–433 28514000 10.1002/jhbp.465

[22] Ignjatovic I, Knezevic S, Knezevic D et al (2017) Standard versus extended lymphadenectomy in radical surgical treatment for pancreatic head carcinoma. J BUON 22:232–238 28365959

[23] Zhang Z, Jia G, Pan G et al (2022) Comparison of the diagnostic efficacy of (68) Ga-FAPI-04 PET/MR and (18)F-FDG PET/CT in patients with pancreatic cancer. Eur J Nucl Med Mol Imaging 49:2877–2888 35243518 10.1007/s00259-022-05729-5

[24] Moon D, Kim H, Han Y et al (2022) Preoperative carbohydrate antigen 19 – 9 and standard uptake value of positron emission tomography-computed tomography as prognostic markers in patients with pancreatic ductal adenocarcinoma. J Hepatobiliary Pancreat Sci 29:1133–1141 33063453 10.1002/jhbp.845

[25] Lee JW, Kang CM, Choi HJ et al (2014) Prognostic Value of Metabolic Tumor Volume and Total Lesion Glycolysis on preoperative (1)(8)F-FDG PET/CT in patients with pancreatic Cancer. J Nucl Med 55:898–904 24711649 10.2967/jnumed.113.131847

[26] Lee W, Oh M, Kim JS et al (2021) Metabolic activity by FDG-PET/CT after neoadjuvant chemotherapy in borderline resectable and locally advanced pancreatic cancer and association with survival. Br J Surg 109:61–70 34378010 10.1093/bjs/znab229

[27] Kawase T, Yasui Y, Nishina S et al (2015) Fibroblast activation protein-alpha-expressing fibroblasts promote the progression of pancreatic ductal adenocarcinoma. BMC Gastroenterol 15:109 26330349 10.1186/s12876-015-0340-0 PMC4556412

[28] Pang Y, Zhao L, Shang Q et al (2022) Positron emission tomography and computed tomography with [(68)Ga]Ga-fibroblast activation protein inhibitors improves tumor detection and staging in patients with pancreatic cancer. Eur J Nucl Med Mol Imaging 49:1322–1337 34651226 10.1007/s00259-021-05576-w

