# Supplementary material for: Prospective Comparison of [18F]FDG and [18F]AIF-FAPI-74 PET/CT in the Evaluation of Potentially Resectable Pancreatic Ductal Adenocarcinoma
Source: Mol Imaging Biol. 2024 Oct 4;26(6):1068–77. doi: 10.1007/s11307-024-01950-w (PMC11634952; doi:10.1007/s11307-024-01950-w)
Supplement: Supplementary file 1 — (MOESM1 1.21 MB) [file 11307_2024_1950_MOESM1_ESM.docx]

**Supplemental material for**

**Prospective Comparison of [^18^F]FDG and [^18^F]AIF-FAPI-74 PET/CT in the Evaluation of Potentially Resectable Pancreatic Ductal Adenocarcinoma**

**Authors:** Won-Gun Yun^1,*^, Joonhyung Gil^2,3,*^, Hongyoon Choi^2,3,4^, Youngmin Han^1^, Hye-Sol Jung^1^, Young Jae Cho^1^, Minseok Suh^2,3^, Wooil Kwon^1^, Yun-Sang Lee^2,3,4^, Gi Jeong Cheon^2,5,6,†^, and Jin-Young Jang^1,†^

**Affiliations: ^a^**Department of Surgery and Cancer Research Institute, Seoul National University College of Medicine, 101 Daehak-ro, Jongno-gu, Seoul, Republic of Korea.

**^b^**Department of Nuclear Medicine, Seoul National University College of Medicine, 101 Daehak-ro, Jongno-gu, Seoul, Republic of Korea.

**^c^**Department of Nuclear Medicine, Seoul National University Hospital, 101 Daehak-ro, Jongno-gu, Seoul, Republic of Korea.

**^d^**Institute of Radiation Medicine, Medical Research Center, Seoul National University, Seoul, Republic of Korea.

**^e^**Department of Molecular Medicine and Biopharmaceutical Sciences, Graduate School of Convergence Science and Technology, Seoul National University, Seoul, Republic of Korea.

^f^Cancer Research Institute & Institute on Aging, Seoul National University, Seoul, Republic of Korea.

^*^These authors contributed equally to this work as co-first authors.

^†^These authors contributed equally to this work as co-corresponding authors.

**Corresponding author and reprints request to:**

Jin-Young Jang, MD, PhD

Department of Surgery and Cancer Research Institute

Seoul National University College of Medicine

101 Daehak-ro, Jongno-gu, Seoul, Republic of Korea, 03080

Phone: +82-2-2072-2194

Fax: +82-2-741-2194

E-mail: [jangjy4@snu.ac.kr](mailto:jangjy4@snu.ac.kr)

**A shortened title:**

Enhanced clinical staging of pancreatic cancer with [^18^F]AIF-FAPI-74 PET/CT

**Manuscript category:**

Original Article


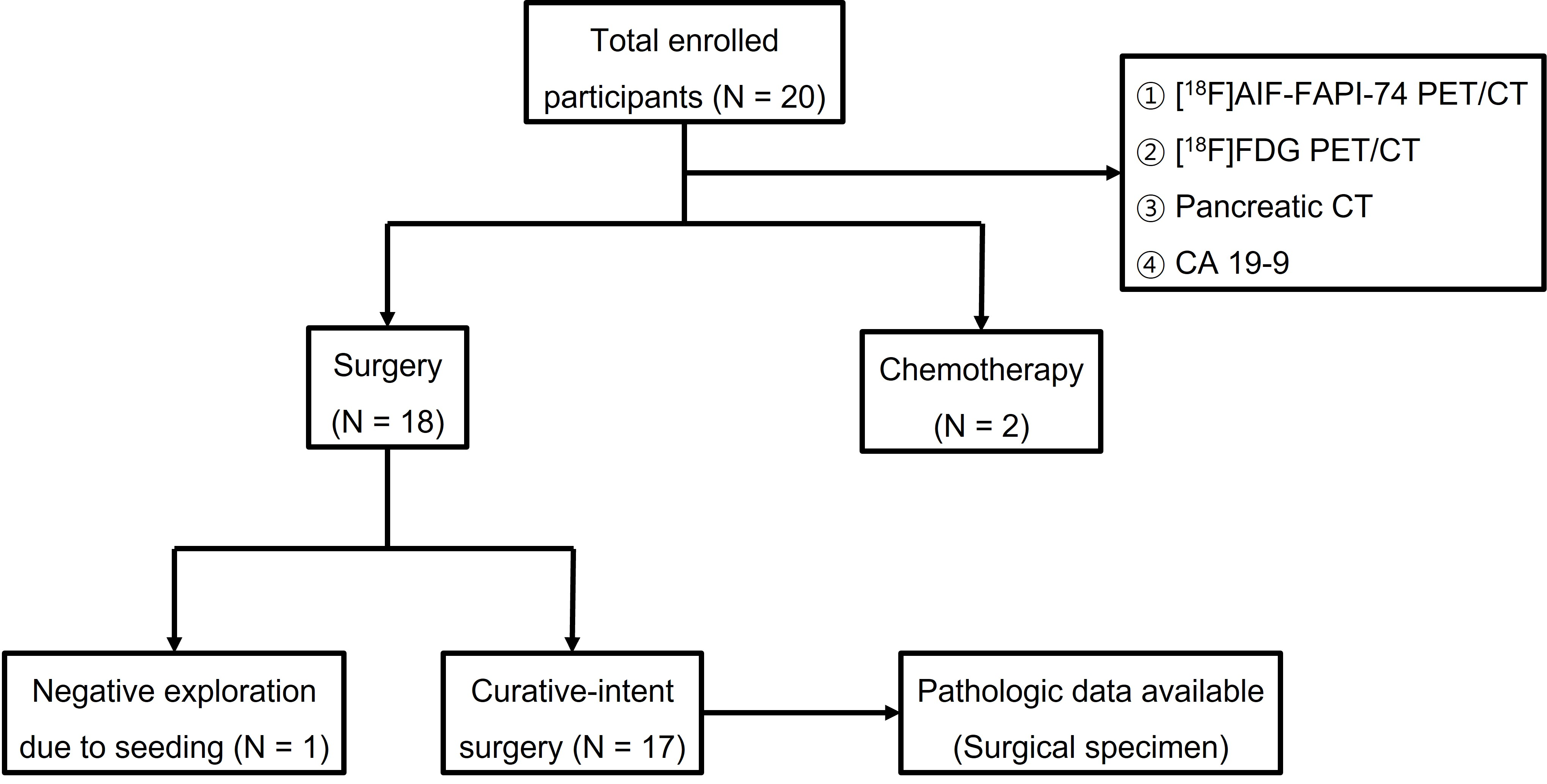


**Figure S1.** Flow diagram of participants enrolled.


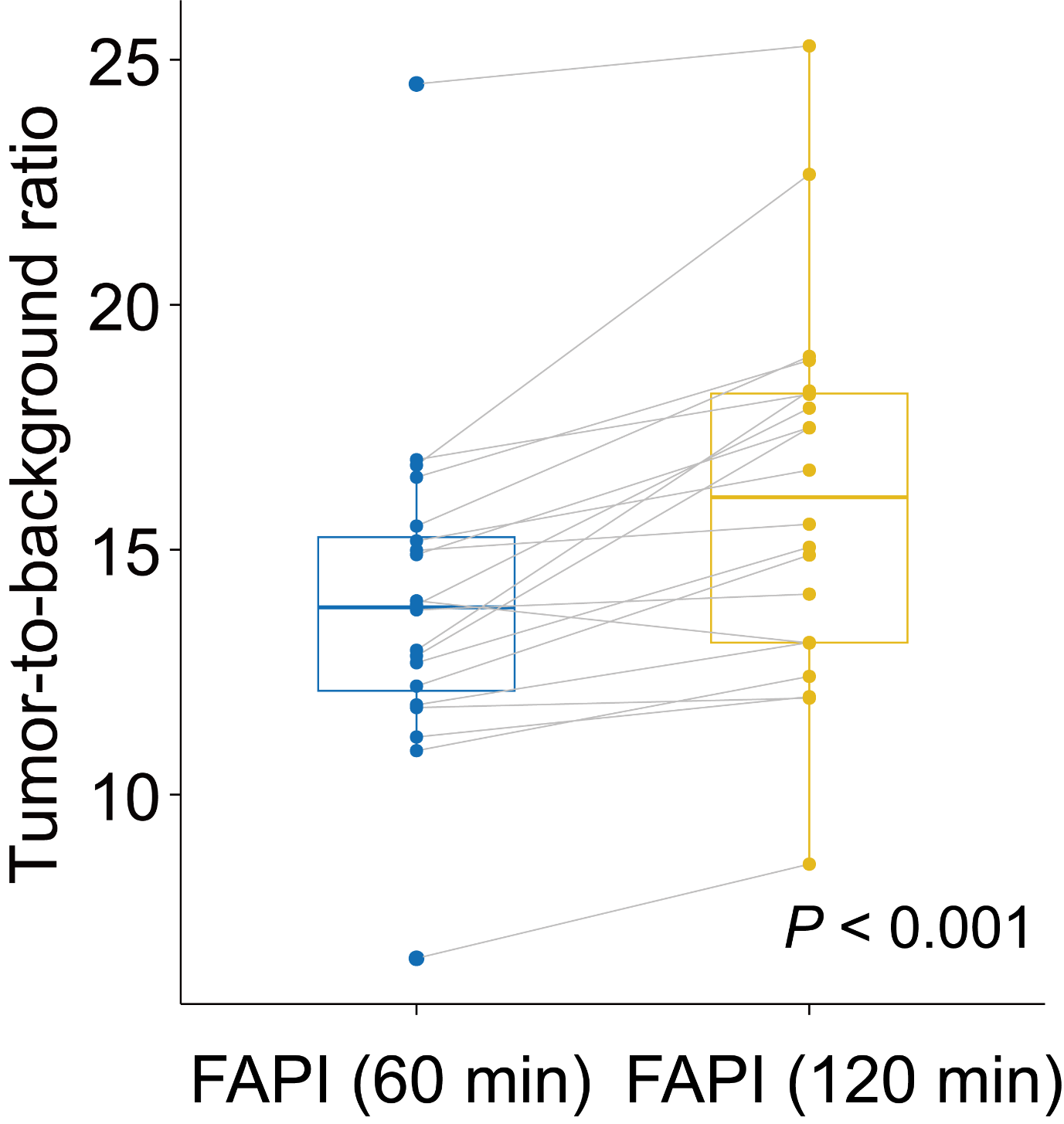


**Figure S2.** Differences of tumor-to-background ratio of primary pancreatic lesions between 60- and 120-minute [^18^F]AIF-FAPI-74 PET/CT.
